# Kyste colobomateux de l'orbite

**DOI:** 10.11604/pamj.2014.17.159.3462

**Published:** 2014-03-05

**Authors:** Hakima Elouarradi, Moulay Zahid Bencherif

**Affiliations:** 1 Université Mohammed V Souissi, Service d’‘Ophtalmologie A de l’'Hôpital des Spécialités, Centre Hospitalier Universitaire, Rabat, Maroc

**Keywords:** Kyste colobomateux, orbite, anomalie congénitale, Colobomatous cyst, orbit, congenital anomaly

## Images in medicine

Le kyste colobomateux de l'orbite est une anomalie congénitale rare, non héréditaire, secondaire à une anomalie de l'embryogénèse par une évagination du feuillet interne de la cupule optique à travers une fente foetale persistante. Cliniquement, Il se présente sous forme d'un petit kyste unilatéral au voisinage du nerf optique, ou parfois un gros kyste envahissant la cavité orbitaire et repoussant au fond de l'orbite un oeil microphtalme, en refoulant en avant la paupière inférieure qui prenait une coloration bleutée. L'oeil contrelatéral peut être normal ou porteur d'une anomalie mineure. Le diagnostic repose sur l’échographie en mode B et le scanner et/ou l'IRM oculo-orbitaire permettant de préciser l’état du globe oculaire et la recherche de lésions associées. L’évolution du kyste peut se faire vers la stabilité ou l'augmentation progressive de sa taille. La conduite thérapeutique dépend de la taille du kyste. Les kystes colobomateux de petite taille seront respectés, avec une surveillance régulière. Les kystes volumineux inesthétiques peuvent être excisés avec réfection de la cavité orbitaire. Il s'agit une fillette de 5 ans, issue d'un mariage consanguin, présente un syndrome malformatif facial. L'examen clinique objective une tuméfaction de la paupière inférieure à droite élastique, fluctuante, non réductible avec une peau en regard fine de coloration bleutée, le globe oculaire non visualisé à l'ouverture de la fente palpébrale. L'oeil gauche est microphtalme (A, B). L'examen général est normal. Le scanner a objectivé la présence de multiples kystes colobomateux orbitaires à droite repoussant en arrière un globe oculaire microphtalme (C, D, E). Notre attitude thérapeutique était l'abstention après explication du pronostic à la famille et la simple surveillance.

**Figure 1:**
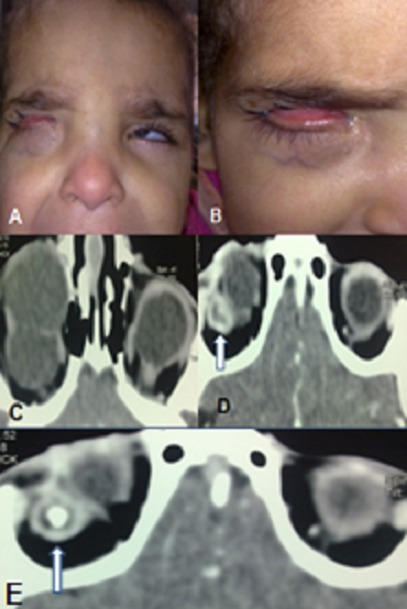
A,B) Tuméfaction de la paupière inférieure droite de coloration bleutée et globe oculaire gauche microphtalme; C,D,E) Coupes scannographiques orbitaires objectivant des kystes colobomoteux envahissant la cavité orbitaire droite et refoulant en arrière un petit globe microphtalme à cristallin calcifié (flèches)

